# SOX2 as a novel contributor of oxidative metabolism in melanoma cells

**DOI:** 10.1186/s12964-018-0297-z

**Published:** 2018-11-22

**Authors:** Elena Andreucci, Silvia Pietrobono, Silvia Peppicelli, Jessica Ruzzolini, Francesca Bianchini, Alessio Biagioni, Barbara Stecca, Lido Calorini

**Affiliations:** 1 Department of Clinical and Experimental Biomedical Sciences “Mario Serio”, Section of Experimental Pathology and Oncology, University of Florence, Viale G.B. Morgagni, 50, 50134 Florence, Italy; 2 Core Research Laboratory, Institute for Cancer Research and Prevention (ISPRO), Florence, Italy; 3 Center of Excellence for Research, Transfer and High Education DenoTHE University of Florence, Florence, Italy

**Keywords:** Melanoma, Tumor extracellular acidosis, SOX2, HIF1α, Oxidative metabolism

## Abstract

**Background:**

Deregulated metabolism is a hallmark of cancer and recent evidence underlines that targeting tumor energetics may improve therapy response and patient outcome. Despite the general attitude of cancer cells to exploit the glycolytic pathway even in the presence of oxygen (aerobic glycolysis or “Warburg effect”), tumor metabolism is extremely plastic, and such ability to switch from glycolysis to oxidative phosphorylation (OxPhos) allows cancer cells to survive under hostile microenvironments. Recently, OxPhos has been related with malignant progression, chemo-resistance and metastasis. OxPhos is induced under extracellular acidosis, a well-known characteristic of most solid tumors, included melanoma.

**Methods:**

To evaluate whether SOX2 modulation is correlated with metabolic changes under standard or acidic conditions, SOX2 was silenced and overexpressed in several melanoma cell lines. To demonstrate that SOX2 directly represses HIF1A expression we used chromatin immunoprecipitation (ChIP) and luciferase assay.

**Results:**

In A375-M6 melanoma cells, extracellular acidosis increases SOX2 expression, that sustains the oxidative cancer metabolism exploited under acidic conditions. By studying non-acidic SSM2c and 501-Mel melanoma cells (high- and very low-SOX2 expressing cells, respectively), we confirmed the metabolic role of SOX2, attributing SOX2-driven OxPhos reprogramming to HIF1α pathway disruption.

**Conclusions:**

SOX2 contributes to the acquisition of an aggressive oxidative tumor phenotype, endowed with enhanced drug resistance and metastatic ability.

**Electronic supplementary material:**

The online version of this article (10.1186/s12964-018-0297-z) contains supplementary material, which is available to authorized users.

## Background

In the last decades, tumor metabolism has drawn increasing attention in the scientific world and deregulating cellular energetics has recently become a hallmark of cancer [1]. Instead of using an oxidative metabolism like most of normal cells, cancer cells convert glucose into lactate even in the presence of high oxygen tension, exploiting the so-called aerobic glycolysis or “Warburg effect”. Despite the energetic gain in terms of ATP production is lower than during the oxidative phosphorylation (OxPhos), the Warburg metabolism is about 100-fold faster than OxPhos and ensures biomass formation and DNA duplication, that is crucial for cancer cell proliferation [2]. Indeed, fermentation to lactic acid and the glycolytic breakdown of glucose generate a number of substrates which turn into “anabolic” precursors for the synthesis of different compounds, such as glucose-6-phosphate for glycogen and ribose 5-phosphate, dihydroxyacetone phosphate for triacylglyceride and phospholipids, and pyruvate for alanine and malate. Metabolite accumulation upstream pyruvate production is further increased by the up-regulation of the low activity M2 isoform of pyruvate kinase (PKM2), that slows down the last step of glycolysis. In this respect, intermediate components of the glycolytic pathway appear to be more significant than its final product pyruvate. Given the limited pyruvate supply, to replenish the tricarboxylic acid cycle (TCA) cancer cells increase glutamine consumption, a key nutrient that provides carbon for acetyl-CoA, citrate production and lipogenesis, nitrogen for purine, pyrimidine and DNA synthesis, and reducing power in the form of NADPH to support cell proliferation [3]. The particular attitude of proliferating cancer cells to use aerobic glycolysis favors a microenvironment enriched in lactate and protons, with a subsequent pH reduction. Moreover, the large amount of lactate released by tumor cells can be taken up by normal stromal cells to regenerate pyruvate, which in turn can be extruded to refuel cancer cells [4]. The reduction in oxygen tension that characterizes proliferating tumor tissues, stimulates the hypoxia-inducible factor α (HIF1α), which drives the anaerobic glycolysis. This leads to lactate dehydrogenase A (LDH-A)-dependent lactic acid production, and the upregulation of monocarboxylated transporter (MCT)-4 and of sodium-proton exporters to avoid intracellular acidosis. As a direct consequence, both aerobic and anaerobic glycolysis adopted by cancer cells contribute to the acidification of tumor microenvironment. Dysregulated pH is emerging as a hallmark of cancer, since cancer cells show a ‘reversed’ pH gradient with a constitutively increased intracellular pH (pHi) that is higher than the extracellular pH (pHe). Indeed, while normal differentiated adult cells show pHi of ∼7.2 and pHe of ∼7.4, cancer cells have a higher pHi (> 7.4) and a lower pHe (6.7–7.1). This ‘reversed’ pH gradient creates a perfect storm for metastatic progression [5] by promoting malignant phenotype endowed with apoptosis resistance, radio- and chemotherapy resistance, immune surveillance escape programs, increased migration and ability of secondary organs colonization [6]. As an additional aspect, we have recently reported that acidic cancer cells undergo a metabolic change characterized by the acquisition of a more OxPhos phenotype through the inhibition of HIF1α expression, associated with a reduced proliferation compared to standard pH condition [7].

Tumor cells are extremely plastic even in terms of cellular energetics and may shift their metabolic phenotypes to adapt to microenvironmental changes, giving a selective advantage to cancer cells under unfavourable environments [8]. Most of solid tumors, including melanoma, undergo such plastic changes in metabolism. Cutaneous melanoma, despite representing less than 5% of all skin cancers, is responsible for the majority of skin cancer-related deaths [9]. The incidence of malignant melanoma in most developed countries has risen faster than any other cancer type since the mid-1950s. It is estimated that the annual increase in the incidence rate of melanoma has been approximately 3–7% per year worldwide for Caucasians. Detection and surgical treatment of early-stage disease seems to prevent progression in most cases. However, patients with deep primary tumors or tumors that metastasize to regional lymph nodes frequently develop distant metastases. Median survival after the onset of distant metastases is only 6–9 months, and the 5-year survival rate is less than 5% [10].

Recent studies have pointed out the crucial role of the transcription factor SOX2 (sex-determining region Y (SRY)-Box2) in melanoma and cancer in general. SOX2 has been correlated with growth, tumorigenicity, drug resistance, and metastasis in at least 25 different tumors, including cancers of the ovary, lung, skin, brain, breast, prostate, and pancreas [11]. In the majority of these cancers, SOX2 has been found to have increased expression or gene amplification in tumor tissues. Moreover, SOX2 has been associated with stemness and tumor initiating cells (TICs), proposed to explain origin and heterogeneity of many tumors [12], including cervical, lung, ovarian, head and neck squamous cell carcinoma, medulloblastoma, skin squamous-cell carcinoma, and melanoma [11]. Indeed, SOX2 has been reported to regulate self-renewal and tumorigenicity of human melanoma-initiating cells [13, 14]. Previous reports indicate that SOX2 is expressed in 50% of melanomas and a minority of nevi [15–17], and is associated with dermal invasion and primary tumor thickness [18]. However, the role of SOX2 in melanoma growth and progression is more controversial. While an early paper reported that SOX2 silencing reduces in vivo growth of A2058 melanoma cells [15], recent studies suggest that SOX2 is dispensable for melanomagenesis and metastasis formation [19, 20].

Here we show for the first time that SOX2 is highly expressed in melanoma cells exposed to extracellular acidosis, where it modulates cell metabolism in order to favor an oxidative phenotype, possibly interfering with HIF1α expression. This additional attitude of SOX2 might add new information on its crucial importance in malignant progression.

## Methods

### Cell cultures

A375-M6 [21], commercial 501-Mel, SK-Mel-2, SK-Mel-5, SK-Mel-28 and patient-derived SSM2c [13] melanoma cell lines were maintained in DMEM 4.5 g/l glucose, 2 mM L-glutamine, and 10% FBS (Euroclone, Milan Italy). 24-h medium acidification was obtained by adding HCl 1 N in complete culture medium to reach pH 6.7 ± 0.1. pH value was monitored by using Orion pH meter 520A-1. pH was monitored for the first hour after medium acidification to check the maintenance of a pH value at 6.7, and then at the end point of each experiment. Cells were treated with 50 mM 2-deoxyglucose (Calbiochem, San Diego, CA, USA) or 10 mM Metformin (Sigma-Aldrich, Milan, Italy) for 24 h.

### *SOX2* silencing and overexpression

*SOX2*-silenced A375-M6 cells were obtained by siRNA transfection with Sox-2 siRNA (sc-38408, Santa Cruz Biotechnology, Dallas, Texas, USA) or control siRNA-A (sc-37007, Santa Cruz Biotechnology), according to manufacturer’s instructions. *SOX2* silencing in SSM2c cells was obtained by lentiviral transduction. Lentiviruses were produced in HEK-293 T cells. Lentiviral vectors used were pLKO.1-puro (LV-c) (Open Biosystems, Lafayette, CO, USA) and pLKO.1-puro-shSOX2–1 (LV-shSOX2–1) targeting the 3′ untranslated region of SOX2 (targeting sequence 5’-CTGCCGAGAATCCATGTATAT-3′) as previously reported [13]. *SOX2* overexpression in 501-Mel cells was obtained by retroviral transduction. Retroviruses were produced in HEK-293 T cells. Retroviral vectors used were generated by co-transfection of 1 μg pBABE (Addgene, Cambridge, MA, USA, #1764) or pBABE-SOX2 (cloned into the BamHI/SalI restriction sites of pBABE vector using the following primers: SOX2-F 5’-ATGTACAACATGATGGAGACGG-3′ and SOX2-R 5’-TCACATGTGTGAGAGGGGC-3′), 0.9 μg pUMVC packaging plasmid (Addgene, #8449) and 0.1 μg pCMV-VSV-G envelope (Addgene, #8454).

### Western blot analysis

Cells were lysed in RIPA buffer (Merck Millipore) containing PMSF (Sigma-Aldrich), sodium orthovanadate (Sigma-Aldrich), and protease inhibitor cocktail (Calbiochem), sonicated and centrifuged 15 min at 14,000 rpm at 4 °C. Equal amounts of protein were separated on Bolt® Bis-Tris Plus gels, 4–12% precast polyacrylamide gels (Life Technologies, Milan, Italy). Fractionated proteins were transferred to a PVDF membrane using the iBlot 2 System (Life Technologies). Following 1-h blocking with Odyssey blocking buffer (Dasit Science, Milan, Italy), membrane was probed overnight at 4 °C with the following primary antibodies: anti-SOX2 mouse monoclonal antibody (R&D System, Minneapolis, MN, USA), anti-HIF-1α rabbit polyclonal antibody (Novusbio, Milan, Italy), anti- GLUT-1, GLUT-3, MCT-1, MCT-4 and PGC1α rabbit polyclonal antibodies (Santa Cruz Biotechnology). After that, membrane was incubated 1 h at room temperature with goat anti-mouse IgG Alexa Fluor 680 antibody (Invitrogen) or goat anti-rabbit IgG Alexa Flour 750 antibody (Invitrogen- Life Technologies, Milan, Italy). Membrane was visualized by the Odyssey Infrared Imaging System (LI-COR® Bioscience, Lincoln, Nebraska USA). Anti-HSP90 (Santa Cruz Biotechnology), β-actin (Sigma-Aldrich) and HDAC2 (Santa Cruz Biotechnology) antibodies were used to assess equal amount of protein loaded in each lane.

### Flow cytometry

Cells were harvested by using Accutase (Euroclone), collected in flow cytometer tubes (2 × 10^5^ cells/tube), permeabilized for 15 min with 0.25% Tryton X-100 PBS, and incubated 1 h at 4 °C with anti-SOX2 antibody (Santa Cruz Biotechnology). Cells were washed in PBS and incubated 1 h in the dark at 4 °C with anti-goat antibody conjugated with FITC (Merk Millipore, Milan, Italy). Samples were washed in PBS and the analyzed at BD FACSCanto (BD Biosciences, Milan, Italy). The flow cytometer was calibrated using cells incubated with secondary antibody only. For each sample, 1 × 10^4^ events were analysed.

### Lactate production

Lactate production by cancer cells was evaluated in 24-h conditioned medium by using D-Lactate Colorimetric Assay Kit (Biovision, CA, USA) according to manufacturer’s instructions. The analysis was performed at the microplate reader (Bio-Rad, Milan, Italy) and data normalized for the cell number of each sample, to get a final result of lactate production (nM) by 1 × 10^5^ cells.

### Glucose uptake detection

Glucose uptake by melanoma cells was evaluated by using Glucose Uptake Cell-Based Assay Kit (Cayman Chemical, Michigan, USA) according to manufacturer’s instructions. Briefly, melanoma cells were glucose-starved for 1 h by using RPMI medium without glucose (Euroclone), then incubated for 15 min in the dark with 2-NBDG, a FITC-labeled deoxyglucose analog, harvested and analyzed at BD FACSCanto (BD Biosciences). The flow cytometer was calibrated using untreated cells. For each sample, 1 × 10^4^ events were analyzed.

### Quantitative real time PCR (qPCR)

Total RNA was prepared using Tri Reagent (Sigma-Aldrich), agarose gel checked for integrity, and reverse transcribed with iScript cDNA Synthesis Kit (Bio-Rad) according to the manufacturer’s instructions. Selected genes were evaluated by a real-time RT-PCR with 7500 Fast Real-Time PCR System (Applied Biosystems, Monza, Italy). Fold change was determined by the comparative Ct method using β-actin, TATA sequence binding protein (TBP), glyceraldehyde 3-phosphate dehydrogenase (GAPDH) and β2-microglobulin as housekeeping genes. Amplification was performed with the PCR setting: 40 cycles of 95 °C for 15 s and of 60 °C for 60 s using PowerUp SYBR Green Master Mix (Thermo Fisher Scientific). Primer sequences (IDT, Tema Ricerca, Bologna, Italy) are listed in Table 1.

**Table 1:** List of forward and reverse primers used for qPCR analysis

Gene	Forward	Reverse
GLUT1	CGGGCCAAGAGTGTGCTAA	TGACGATACCGGAGCCAATG
GLUT3	CGAACTTCCTAGTCGGATTG	AGGAGGCACGACTTAGACAT
HK2	CAAAGTGACAGTGGGTGTGG	GCCAGGTCCTTCACTGTCTC
LDHA	AGGGAATGTACGGCATTGAG	CCTCATCGTCCTTCAGCTTC
PDK1	CCAAGACCTCGTGTTGAGACC	AATACACGTCTCAGGTCTCCTTGG
PDP2	TAGGCCAACCTTTGTTTCACCA	AGACCCTCACAACAAAAGCCT
MCT-1	GTGGCTCAGCTCCGTATTGT	GAGCCGACCTAAAAGTGGTG
MCT-4	CAGTTCGAGGTGCTCATGG	ATGTAGAGGTGGGTCGCATC
PGC1α	GGGAAAGTGAGCGATTAGTTGAG	CATGTAGAATTGGCAGGTGGAA
CytC	TTGCACTTACACCGGTACTTAAGC	ACGTCCCCACTCTCTAAGTCCAA
COX4I	GGCCCGGCATTTTACGA	TCACCGTGGAGCGGAAA
COX5b	TGCGCTCCATGGCATCA	CCCAGTCGCCTGCTCTTC
ATP5A1	TGCAAGGACTTCCATGCCTC	CGCCCAGGTTCTTCAAGATCAA
SOX2	GAGCTTTGCAGGAAGTTTGC	GCAAGAAGCCTCTCCTTGAA
HIF1a promoter	TGCAAAGTTGCCAAAGGCCA	CAGGGGAACTCACCTTGTCTAC
HIF1a	GGCGCGAACGACAAGAAAAA	TCCAAATCACCAGCATCCAGA
TBP	CAACAGCCTGCCACCTTAC	CTGAATAGGCTGTGGGGTC
ACTIN	TCGAGCCATAAAAGGCAACT	CTTCCTCAATCTCGCTCTCG
GAPDH	GACGCTGGGGCTGGCATTG	GCTGGTGGTCCAGGGGTC
Β2-microglobulin	GCCGTGTGAACCATGTGACT	GCTTACATGTCTCGATCCCACTT

### Annexin V/PI flow cytometer analysis

Cell death was determined by flow cytometer analysis using Annexin V FITC-conjugated (Immunotools GmbH, Friesoythe, Germany) and PI (Sigma-Aldrich) according to the manufacturer’s protocol. Briefly, cells were harvested with Accutase (Eurolone), collected in flow cytometer tubes (1 × 10^5^ cells/tube), washed in PBS, and incubated 15 min at 4 °C in the dark with 100 μl Annexin binding buffer (100 mM HEPES, 140 mM NaCl, 25 mM CaCl2, pH 7.4), 1 μl of 100 μg/ml PI working solution, and 5 μl Annexin V FITC-conjugated. Each sample was added with Annexin binding buffer to reach 500 μl volume/tube. Samples were analyzed at BD FACSCanto (BD Biosciences). Cellular distribution depending on Annexin V and/or PI positivity allowed the measure of the percentage of viable cells (Annexin V and PI negative cells), early apoptosis (Annexin V-positive and PI negative cells), late apoptosis (Annexin V and PI-positive cells), and necrosis (Annexin V-negative and PI-positive cells).

### Chromatin immunoprecipitation

Melanoma cells were fixed with 1% formaldehyde for 10 min and lysed in cell Lysis Buffer (5 mM PIPES pH 8, 85 mM KCl, 0.5% NP-40) added with protease inhibitors. Nuclei were collected by centrifugation at 4500 rpm for 10 min and lysed in nuclear lysis buffer (1% SDS, 10 mM EDTA, 50 mM Tris-HCl pH 8) added with protease inhibitors. Chromatin was sonicated to an average size of 200–600 bp, diluted with ChIP Dilution Buffer (10 mM Tris-HCl pH 8, 1% Triton X-100, 2 mM EDTA, 140 mM NaCl) and incubated overnight with 20 μl of protein G magnetic dynabeads pre-conjugated with mouse anti-SOX2 (MAB2018; R&D System) or normal mouse IgG (sc-2025; Santa Cruz Biotechnology) antibodies. DNA was purified and qPCR was carried out at 60 °C using FastStart SYBR Green Master (Roche Diagnostic, Monza, Italy) in a Rotorgene-Q (Qiagen, Milan, Italy). Primer sequences are listed in Table 1.

### Luciferase reporter assays

Luciferase reporters were used in combination with Renilla luciferase pRL-TK reporter vector (Promega, Madison, WI) to normalize luciferase activities. pCS2 + MT vector (Promega) was used to equal DNA amounts; pCS2 + SOX2 was cloned into pCS2 + MT using the following primers: Fwd 5’-ATGTACAACATGATGGAGACGG-3′, Rev. 5’-CACATGTGTGAGAGGGGC-3′ after digestion with XhoI/SnaBI restriction enzymes; pGL4.20-HIF1αprom was purchase from Addgene (Plasmid #40173). Luminescence was measured using the Dual-Glo Luciferase Assay System (Promega) and the GloMax® 20/20 Luminometer (Promega).

### Statistical analysis

The experiments were performed at least three times for a reliable application of statistics. Statistical analysis was performed with GraphPad Prism software. Values are presented as mean ± SD. ANOVA or Student’s T test were used to evaluate the statistical significance.

## Results

### Extracellular acidosis promotes SOX2 expression in melanoma cells contributing to OxPhos metabolism

**Fig. 1:**
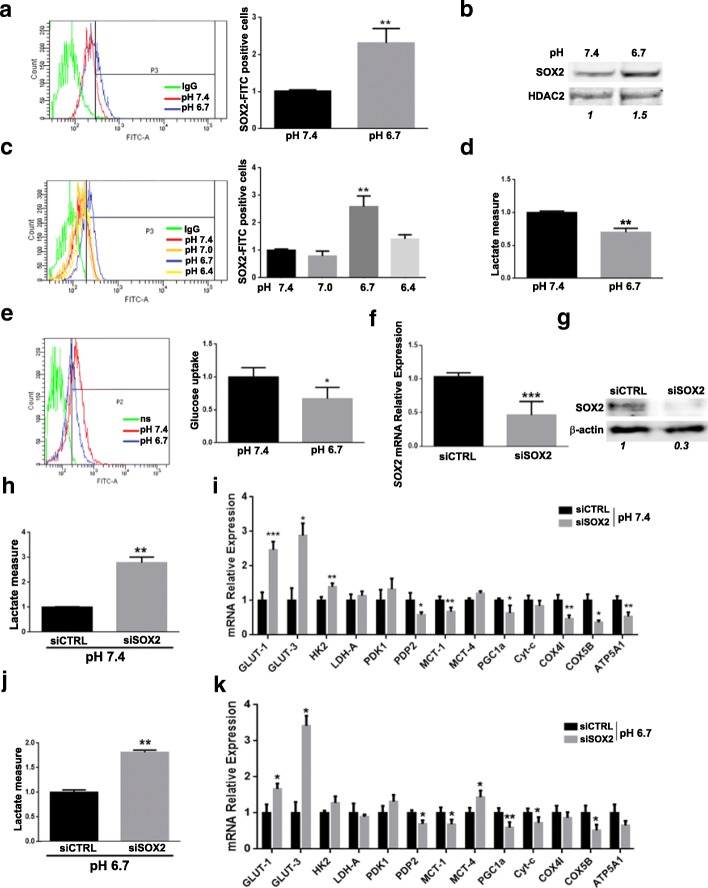
SOX2 is up-regulated by extracellular acidosis and its inhibition increases the glycolytic metabolism in A375-M6 melanoma cells. **a**, **b**) Representative flow cytometry plot (left) and relative quantification chart (right) (**a**) and Western blot (**b**) of SOX2 in A375-M6 cells exposed for 24 h to standard pH 7.4 and acidic pH 6.7. *p* < 0.05, T-test. *N* = 3. HDAC2 was used as loading control of nuclear protein fraction. Quantification of SOX2 protein expression in shown in italic. **c**) Representative flow cytometry plot (left) and relative quantification chart (right) of SOX2 level variation along with pH values. *p* < 0.01, T-test, *N* = 3. **d**) Quantification of lactate production by A375-M6 cells exposed for 24 h to standard (pH 7.4) or acidic (pH 6.7) conditions. *p* < 0.01, T-test, *N* = 3. **e**) Representative flow cytometry plot (left) and quantification of glucose uptake (right) in A375-M6 cells exposed for 24 h to standard (pH 7.4) or acidic (pH 6.7) conditions. *p* < 0.05, T-test, N = 3. **f**, **g**) Quantitative Real Time PCR (qPCR) (**f**) and Western blot (**g**) of SOX2 in A375-M6 silenced for SOX2 (siSOX2) compared to control (siCTRL). Quantification of SOX2 protein is shown in italic. β-actin used as loading control. *p* < 0.01, T-test. *N* = 3. **h**) Quantification chart of lactate production by A375-M6 siSOX2 compared to siCTRL in standard condition (pH 7.4) *p* < 0.01, T-test, *N* = 3. **i**) qPCR of a panel of glycolysis- and OxPhos-related genes of A375-M6 in standard condition (pH 7.4) silenced for *SOX2* (siSOX2) compared to control (siCTRL). *p < 0.05, **p < 0.01; ***p < 0.001, T-test, *N* = 3. **j**) Quantification chart of lactate production by acidosis-exposed (pH 6.7) siSOX2 A375-M6 compared siCTRL. *p* < 0.01, T-test, *N* = 3. **k**) qPCR analysis of a panel of glycolysis- and OxPhos-related genes of acidosis-exposed (pH 6.7) A375-M6 siSOX2 compared siCTRL. *p < 0.05, **p < 0.01, ***p < 0.001, T-test. *N* = 3

We have previously investigated the role of acidic tumor microenvironment in the acquisition of an aggressive [22] and chemo-resistant [23] phenotype in melanoma cells and found a closely related metabolic reprogramming that switches acidic cells to a more oxidative metabolism [7]. In this context, we hypothesized that the expression of the transcription factor SOX2 might be modulated by pH variations of tumor extracellular microenvironment and have a role in this metabolic adaptation. Flow cytometry and western blot analyses (Fig. 1a and b) showed increased SOX2 expression in A375-M6 melanoma cells exposed to acidic medium (pH 6.7) compared to control (pH 7.4). We also observed that SOX2 expression was strictly regulated by pH variations: while pH 7.0 was not sufficient to increase SOX2 expression, we obtained a maximal level at pH 6.7, but further medium acidification up to pH 6.4 restored SOX2 expression at control level (Fig. 1c). As previously reported [7], extracellular acidosis induces a metabolic shift towards OxPhos with a simultaneous slowdown of the glycolytic pathway, as confirmed by the reduced lactate production (Fig. 1d) and glucose uptake (Fig. 1e) by acidosis-exposed melanoma cells compared to control. To understand whether SOX2 up-regulation in acidic melanoma cells might be implicated in the reconversion to OxPhos metabolism, *SOX2* was silenced (Fig. 1f and g). Lowering *SOX2* level in melanoma cells, besides causing a decrease in cell proliferation (Additional file 1: Figure S1a) in accordance with the literature [13], correlated with a more glycolytic metabolism, as confirmed by augmented lactate production (Fig. 1h) and the increment of glycolytic gene expression together with a reduction of oxidative ones (Fig. 1i and Additional file 1: Figure S2). This change in metabolism elicited by *SOX2* silencing was also evident in melanoma cells exposed to extracellular acidosis (pH 6.7) (Fig. 1j and k), confirming the contribution of SOX2 to the OxPhos metabolic adaptation. Indeed, *SOX2* silencing in melanoma cells, under both standard and acidic conditions, favored an enhanced expression of glucose transporters *GLUT-1* and *GLUT-3*. Further, hexokinase isoform 2 (*HK2*), which catalyzes the rate-limiting first step of glycolysis, was stimulated upon *SOX2* silencing in either standard or acidic cells, although in acidic cells does not reach a significantly level (Fig. 1i and Additional file 1: Figure S2). To evaluate the activity of pyruvate dehydrogenase (*PDH*) enzyme, which drives the pyruvate enter into TCA cycle linking glycolysis to OxPhos, we tested the expression of two enzymes that regulate *PDH*: the activating pyruvate dehydrogenase phosphatase 2 (*PDP2*) and the inhibiting pyruvate dehydrogenase kinase 1 (*PDK1*). In *SOX2*-silenced control and acidic melanoma cells, we observed reduction of *PDP2* and an appreciable increase of *PDK1* (Fig. 1i and k), suggesting an impaired mitochondrial OxPhos. Crucial for acidosis-adapted tumor cells, which have reprogrammed their metabolic phenotype to OxPhos, is the up-regulation of monocarboxylate transporter 1 (*MCT1*), a promoter of lactate influx. Instead, tumor cells prevalently relying on Warburg metabolism up-regulate monocarboxylate transporter 4 (*MCT4*) expression, a promoter of lactate efflux, in order to prevent intracellular acidification and subsequent cell death. We observed a *MCT1* reduction and *MCT4* increase in *SOX2*-depleted melanoma cells (Fig. 1i and k). Despite significant variations were obtained only under acidic condition, we believe that *MCT1* reduction with an unchanged level of *MCT4* observed in standard condition might be also indicative of a net lactate discharge. A dynamic test of lactate production (Fig. 1j) confirmed that *SOX2* silencing in acidosis-exposed melanoma cells reverted OxPhos to a more glycolytic metabolism, further confirmed by the down-regulation of OxPhos-related enzymes, peroxisome proliferator-activated receptor gamma coactivator 1-α (*PGC1α*), cytochrome-c (*Cyt-c*), cytochrome c oxidase subunit 4 isoform 1 (*COX4I*), cytochrome c oxidase subunit 5B (*COX5B*), ATP Synthase F1 Subunit Alpha (*ATP5A1*) (Fig. 1k). Reduction of the *PGC1α* in both control and acidic-exposed melanoma cells is of special importance considering its activity in mitochondrial biogenesis and respiration in cancer cells tightly related to malignancy [24].

### Metabolic drugs sustain SOX2 contribution to OxPhos metabolism in acidic A375-M6 melanoma cells

**Fig. 2:**
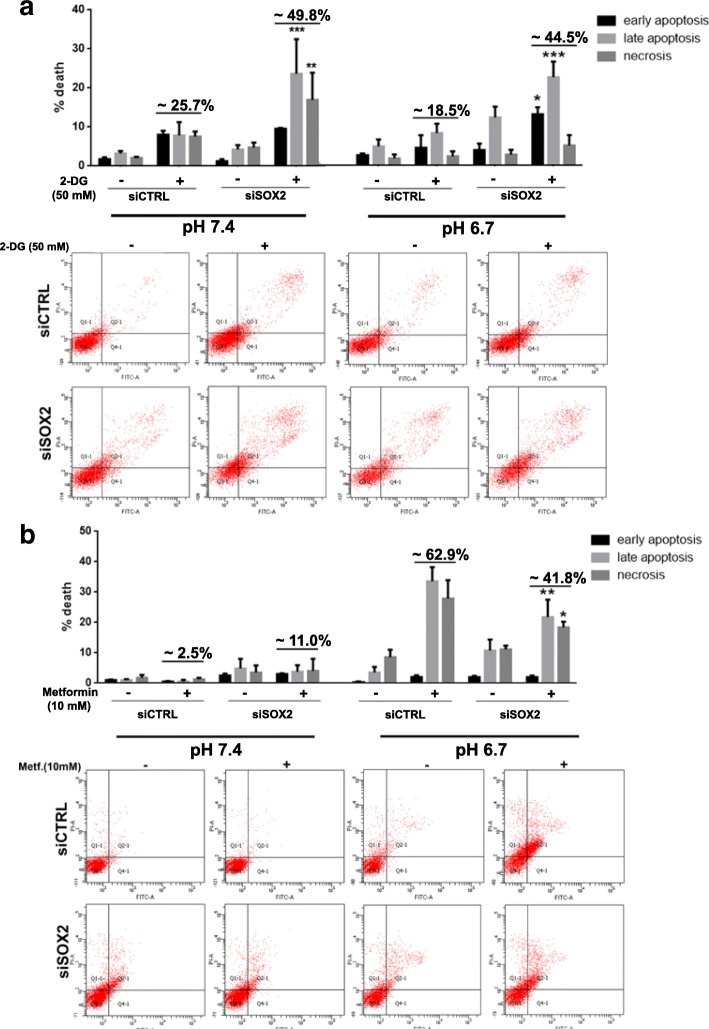
*SOX2* silencing in A375-M6 melanoma cells alters the efficacy of the metabolic drugs 2-DG and Metformin. **a**) Quantification chart (upper panel) and representative pictures (lower panel) of Annexin V/PI analysis of A375-M6 treated for 24 h with the anti-glycolytic drug 2-DG (50 mM) under standard (pH 7.4) or acidic (pH 6.7) conditions. **b**) Quantification chart (upper panel) and representative pictures (lower panel) of Annexin V/PI analysis of A375-M6 treated for 24 h with the anti-OxPhos drug Metformin (10 mM) under standard (pH 7.4) or acidic (pH 6.7) conditions. *p < 0.05, **p < 0.01, ***p < 0.001, Two-way ANOVA (statistical analysis compares for each phase- early apoptosis, late apoptosis, and necrosis- siSOX2 versus the respective untreated or treated siCTRL). *N* = 3

To further confirm the metabolic effects of *SOX2* downregulation, we tested the efficacy of two metabolic drugs in control and *SOX2*-silenced A375-M6 melanoma cells maintained in standard condition (pH 7.4) or exposed to extracellular acidosis (pH 6.7). We used 2-Deoxy-D-glucose (2-DG, 50 mM), a glucose analog which competitively inhibits glucose uptake and blocks the first critical step of glucose metabolism [25], and the mitochondrial respiration poison Metformin, a well-known antidiabetic drug that targets the complex I of the respiratory chain [26]. Treatment with 2-DG predisposed control A375-M6 grown in standard condition (pH 7.4) to cell death (~ 25%), but these cytotoxic effects were almost doubled in siSOX2 melanoma cells (~ 45% cell death), suggesting their enhanced glycolytic dependency (Fig. 2a). On the contrary, Metformin treatment did not affect cell viability neither in control nor in siSOX2 cells (~ 2.5 and 11% cell death, respectively), demonstrating insensitiveness to OxPhos inhibitors (Fig. 2a and b, pH 7.4). Upon melanoma cell exposure to extracellular acidosis, 2-DG exerted less cytotoxic effects in control cells (~ 18.5%) compared to what observed under standard pH condition, but induced a rapid enhancement of cell death in siSOX2 cells (~ 44.5%), disclosing a reverted attitude of these cells to glycolytic metabolism. On the other hand, Metformin was effective on acidic control cells (~ 62.9% cell death), as expected, but its cytotoxic effects were significantly reduced in acidic siSOX2 cells (~ 41.8%), highlighting a reduced OxPhos metabolism in these cells (Fig. 2a and b, pH 6.7). These results are consistent with our hypothesis that *SOX2* down-regulation promotes a glycolytic metabolism, sensitizing melanoma cells to 2-DG and, at the same time, reducing the cytotoxic effect of Metformin.

### Modulation of SOX2 expression and metabolic adaptation of SSM2c and 501-Mel melanoma cells under standard conditions

**Fig. 3:**
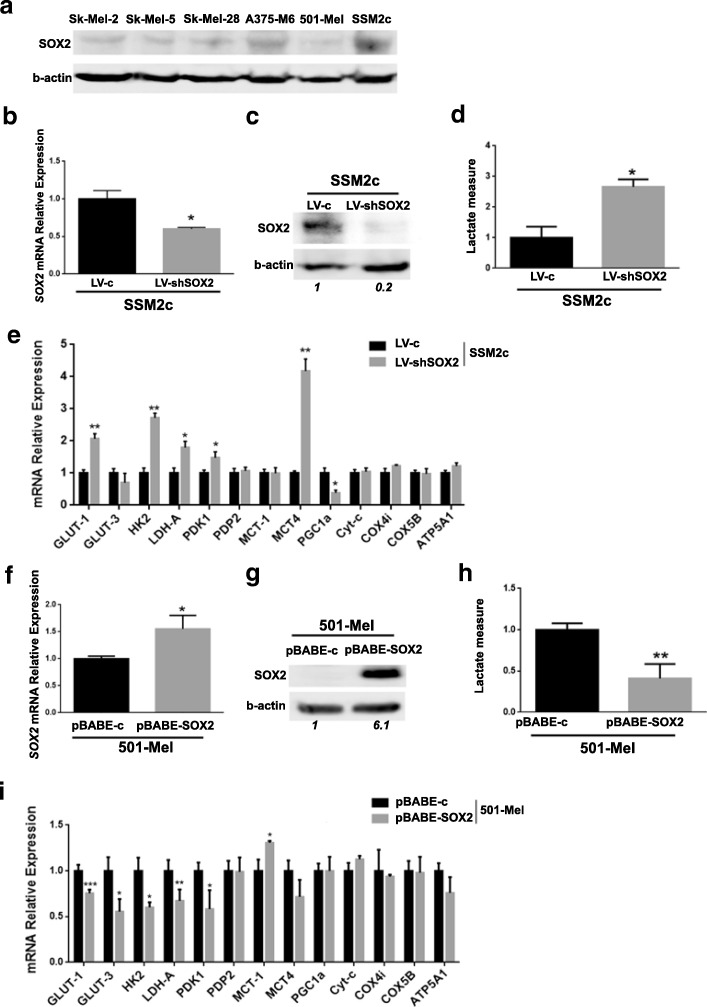
Modulation of SOX2 expression induces metabolic changes in SSM2c and in 501-Mel melanoma cells. **a**) Western blot analysis of SOX2 expression in SK-MEL-2, SK-MEL-5, SK-MEL-28, A375-M6, 501-Mel and SSM2c melanoma cells. β-actin was used as loading control. **b**, **c**) qPCR (**b**) and Western blot (**c**) of SOX2 in SSM2c silenced for SOX2 (LV-shSOX2) compared to control (LV-c). Quantification of SOX2 protein is shown in italic. *p* < 0.05, T-test. *N* = 3. **d**) Quantification chart of lactate production of SSM2c LV-shSOX2 compared to LV-c. *p* < 0.05, T-test, *N* = 3. **e**) qPCR of a panel of glycolysis- and OxPhos-related genes in SSM2c LV-shSOX2 compared to LV-c. *p < 0.05, **p < 0.01, ***p < 0.001, T-test. *N* = 3. **f**, **g**) qPCR (**f**) and Western blot (**g**) of SOX2 in 501-Mel with SOX2 overexpression (pBABE-SOX2) compared to control (pBABE-c). Quantification of SOX2 protein is shown in italic. *p* < 0.01, T-test. *N* = 3. **h**) Quantification chart of lactate production of 501-Mel pBABE-SOX2 compared to pBABE-c. *p* < 0.01, T-test, N = 3. **i**) qPCR of a panel of glycolysis- and OxPhos-related genes in 501-Mel pBABE-SOX2 compared to pBABE-c. *p < 0.05, **p < 0.01, ***p < 0.001, T-test. *N* = 3

To confirm the correlation between SOX2 and OxPhos metabolism and evaluate the occurrence of any metabolic variations, we modulated *SOX2* expression in other two human melanoma cell lines, chosen among a panel of cell lines based on their SOX2 expression level: patient-derived SSM2c cells, that express high levels of SOX2, and 501-Mel cells, that express very low SOX2 levels (Fig. 3a). When *SOX2* was silenced in high SOX2-expressing SSM2c cells (Fig. 3b and c), besides a decrease in cell proliferation (Additional file 1: Figure S1b), we observed a significant increase of lactate production (Fig. 3d) and of glycolytic genes, such as *GLUT-1*, *HK2*, lactate dehydrogenase A (*LDH-A*), *PDK-1*, *MCT-4*, coupled with a *PGC1α* reduction (Fig. 3e and Additional file 1: Figure S2). Instead, when low SOX2-expressing 501-Mel were forced to express SOX2 (Fig. 3f and g), we observed, along with increased cell proliferation (Additional file 1: Figure S1c), a decreased release of lactate in their media (Fig. 3h) and down-regulation of glycolytic genes, such as *GLUT-1*, *GLUT-3*, *HK2*, *LDH-A*, *PDK-1*, (Fig. 3i and Additional file 1: Figure S2). Furthermore, ectopic SOX2 expression in 501-Mel increased the expression of *MCT-1*, which generally correlates with oxidative metabolism, but did not affect the other OxPhos-related genes (Fig. 3i and Additional file 1: Figure S2). All together, these findings confirm the role of SOX2 in OxPhos control.

### SOX2-driven metabolic adaptation to OxPhos is due to HIF1α pathway disruption

**Fig. 4:**
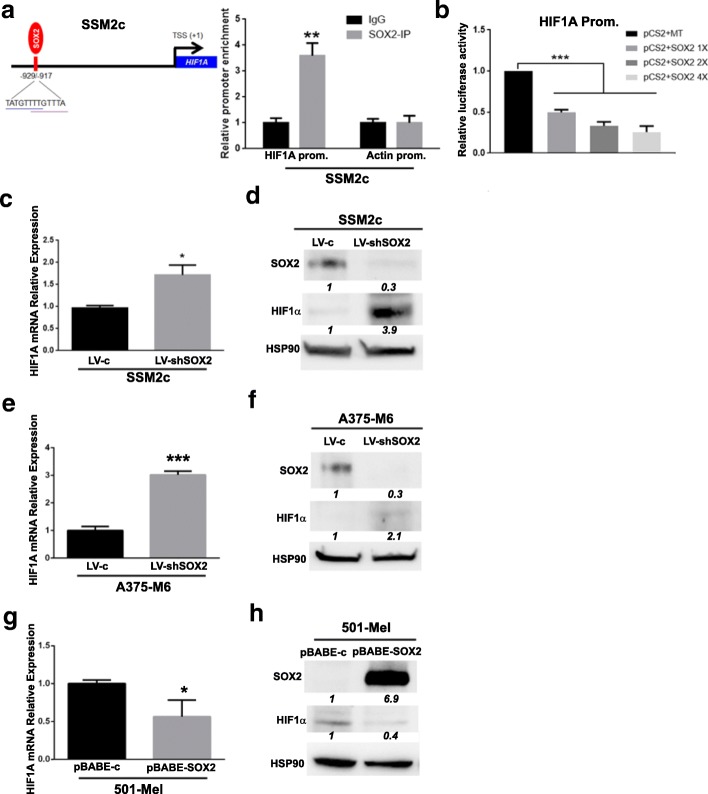
SOX2 regulates HIF1α expression in SSM2c, A375-M6 and 501-Mel melanoma cells. **a**) (Left) Representative image of *HIF1A* promoter showing the sequence and the position of the putative *SOX2*-binding sites (BS) relative to the transcriptional start site (TSS). (Right) Chromatin Immunoprecipitation (ChIP) assay showing *SOX2* binding at *HIF1A* promoter; *Actin* promoter was used as negative control and set to 1. *p* < 0.01, Two-way ANOVA. **b**) Quantification of dual-luciferase reporter assay in SSM2c cells. Relative luciferase activities were Firefly/Renilla ratios, with the level induced by control equated to 1. Data represent mean ± s.e.m. *p* < 0.001 vs control, One-way ANOVA. *N* = 4. **c**, **d**) qPCR (**c**) and Western blot (**d**) of HIF1α in SSM2c LV-shSOX2 compared to LV-c. Quantification of SOX2 and HIF1α protein is shown in italic. *p* < 0.01, T-test. *N* = 4. **e**, **f**) qPCR (**e**) and Western blot (**f**) of HIF1α in A375-M6 LV-shSOX2 compared to LV-c. Quantification of SOX2 and HIF1α protein is shown in italic. *p* < 0.05, T-test. *N* = 4. **g**, **h**) qPCR (**g**) and Western blot (**h**) of HIF1α in 501-Mel pBABE-SOX2 compared to pBABE-c. Quantification of SOX2 and HIF1α protein is shown in italic. *p* < 0.01, T-test. *N* = 4. HSP90 was used as loading control

Given the results obtained after modulation of SOX2 expression in melanoma cell lines grown in acidic and standard conditions, we hypothesized the involvement of HIF1α transcription factor in SOX2-driven metabolic adaptation. Indeed, our findings indicate that low levels of SOX2 correlate with a blockage of the TCA cycle mediated by either a down-regulation of *PDP2* or an up-regulation of *PDK1* genes that, coupled with a higher lactate production and the modulation of *GLUTs*, *HK2*, *LDH-A* and *MCTs* genes, lead to the switch toward anaerobic glycolysis. In addition, we already know that melanoma cell exposure to extracellular acidosis deeply inhibits HIF1α expression [21], leaving tumor cells to acquire an OxPhos phenotype, probably under the influence of the increased SOX2 expression, and able to maximize energy efficiency with the available resources. Adaptation to extracellular acidosis elicits a reduction of the energy sensor AMP-activated protein kinase (AMPK), which inhibits anabolic pathways and induces cell cycle slow down, dampening ATP consumption [7]. Acidosis-driven HIF1α inhibition may suggest that, in certain circumstances, acidosis more than hypoxia could have a role in malignant progression. To confirm our hypothesis, we performed a bioinformatic analysis for the presence of putative *SOX2*-binding sites (BS) in the *HIF1A* promoter, and found two overlapping putative SOX2 BS in a region encompassing − 929/− 917 bp upstream the transcription start site (TSS) (Fig. 4a). Chromatin Immunoprecipitation (ChIP) showed that SOX2 binds to the *HIF1A* promoter in that region (Fig. 4a), indicating that *HIF1A* is a direct downstream target of *SOX2*. To determine the effect of SOX2 binding on *HIF1A* promoter, SOX2 was transfected with the *HIF1A* promoter (− 2159 bp/+ 49 bp) containing the putative SOX2-BS driven by a luciferase reporter. Luciferase assay showed that SOX2 decreased the activity of the reporter in a dose-dependent manner (Fig. 4b), indicating that SOX2 inhibits HIF1α expression by directly binding to its promoter. Thus, it was reasonable to evaluate if any HIF1α variations occurred in response to SOX2 modulation in our cellular models. SOX2 silencing in SSM2c and A375-M6 increased the expression of HIF1α mRNA and protein (Fig. 4c-f). Consistently, overexpression of SOX2 in 501-Mel melanoma cells drastically reduced HIF1α mRNA and protein expression (Fig. 4g and h). These results indicate that SOX2 negatively regulates HIF1α in melanoma cells. It should be considered that such variations have been observed under normoxic conditions, when HIF1α is generally not completely stabilized. These HIF1α variations might be enough for the metabolic changes observed in our experimental models of SOX2-manipulated melanoma cells.

## Discussion

Cancer cells are characterized by a deregulated metabolism since, unlike normal cells, they largely depend on glycolysis even in the presence of oxygen, a phenomenon referred to as “Warburg effect” or aerobic glycolysis [3]. Nevertheless, cancer metabolism does not exclusively depend on aerobic glycolysis. Indeed, tumor cells can rather shift between different metabolic phenotypes or be in a hybrid state utilizing both glycolytic and oxidative metabolism [27]. This plasticity is also referred to cancer bioenergetics and contributes to positively select cancer cells in order to survive besides any environmental changes and hostile conditions [8]. We and others have already reported that the acidic microenvironment, that characterizes most of solid tumors and is associated with aggressive tumor phenotypes [22], favors OxPhos at the expense of glycolysis [7, 28–32]. Here we correlate for the first time SOX2 expression in acidic melanoma cells with a more oxidative metabolism, that is in turn associated with tumor progression and poor prognosis. In this regard, OxPhos metabolism has recently regained its role in cancer progression, given its association with occurrence of chemo-resistance and development of metastasis [27]. Moreover, despite the existence of controversial opinions [33], recent studies suggest that cancer stem cells are more reliant upon an oxidative metabolism than the non-stem bulk in different tumor types, including leukemia, ovarian, pancreatic, and breast cancer. OxPhos metabolism has been also shown to be privileged by circulating tumor cells compared to primary tumor cells of melanoma and breast cancers [24], and to be correlated with chemo-resistance in glioma [34], lung [35], pancreatic [36]**,** prostate [37], and ovarian cancers [38]. Furthermore, several cases of metabolic shift to OxPhos following targeted therapies have been reported [39]. This is the case of melanomas carrying activating *BRAF* mutations, where *BRAF* inhibitors induce *PGC1α,* a master regulator of mitochondrial biogenesis, which in turn promotes oxidative metabolism [40].

By exploiting an in vitro model of extracellular acidosis, we demonstrated that SOX2 is induced by an acidic microenvironment and, importantly, that *SOX2* depletion in acidic melanoma cells reprograms their metabolism to a more glycolytic phenotype, also reducing OxPhos-related genes that characterize acidosis-exposed melanoma cells. The reprogramming toward a more glycolytic profile is also evident in *SOX2*-silenced cells grown in standard condition. A tightly correlation between SOX2 and OxPhos emerges when *SOX2*-silenced melanoma cells, either grown in acidic or standard pH medium, are treated with 2-DG and Metformin. 2-DG targets glucose metabolism inducing a decrease of ATP generation, whereas Metformin blocks complex I of the respiratory chain. Interestingly, epidemiological and retrospective studies have revealed a lower incidence of cancer and better outcomes in diabetic patients taking Metformin compared to non-diabetics or diabetics using alternative drugs [41]. We found that 2-DG promotes cell death in *SOX2*-silenced cells grown in standard pH conditions and, most importantly, also in acidic melanoma cells depleted of SOX2. On the other hand, Metformin was effective only in acidosis-exposed cancer cells, since its efficacy is significantly reduced in *SOX2*-silenced cells, even though a further cell death reduction could be expected. This could be probably due to the high levels of SOX2 in acidic melanoma cells associated with an only partial *SOX2* silencing efficacy.

To better understand SOX2 contribution in OxPhos metabolism, we determined metabolic markers in SSM2c cells, characterized by high SOX2 levels, and in 501-Mel, which show low/no SOX2 expression, upon knock-down or ectopic expression of SOX2, respectively. We confirmed the ability of SOX2 to contribute to an oxidative metabolism. Indeed, *SOX2* knock-down leads to the suppression of the master regulator of mitochondrial metabolism *PGC1α,* a phenomenon associated with the promotion of critical steps of the glycolytic pathway, i.e. *GLUTs*, *HK2*, *PDP2*/*PDK1* axis and *LDH-A*. Furthermore, *SOX2* silencing induces a switch of *MCT* genes from type 1 to type 4, indicating a preferred lactate efflux characteristic of a glycolytic metabolism. Consistently, in SOX2 overexpressing 501-Mel cells the reduction of most glycolytic markers came together with a promotion of *MCT-1*, a promoter of lactate influx. The clinical importance of MCT expression levels derived from their tightly correlation with shorter overall survival of advanced melanoma patients [42].

SOX2 has been already associated with tumor initiation, growth, drug resistance, and metastasis. Chemo-resistant cancer cells that appear to preferably exploit oxidative metabolism, have been also associated with enhanced SOX2 expression in gastric, lung, prostate, colorectal [11], and breast [43] cancers. These findings prompted us to verify whether SOX2 influence in cell metabolism might be related to HIF1α activity, considering that HIF1α strongly induces a glycolytic phenotype. Our results indicate that HIF1α and SOX2 are inversely correlated in normoxic condition, and this effect might be functionally sufficient to reprogram melanoma cells toward OxPhos. This is likely true mainly in conditions when SOX2 exceeds HIF1α in terms of protein expression, as in the case of acidosis-exposed melanoma cells. Instead, under hypoxia, HIF1α stabilization, despite the presence of SOX2 [44], likely represents the leading factor that causes cancer cell metabolic switch to anaerobic glycolysis. Among the so-called non-canonical HIF1α regulation [45], it is quite interesting to recall that an increased lactate production is able to promote HIF1α stabilization, although the mechanism has not been yet clarified [46]. Thus, the lactate increase observed in SOX2-silenced acidic and non-acidic melanoma cells could be able to contribute to HIF1α expression and glycolytic re-conversion. Furthermore, quite recently it was demonstrated that HIF1α represses PGC1α expression in renal cell carcinoma, suggesting a regulatory loop among these transcriptional factors, involving oxygen sensing to mitochondrial biogenesis [47]. This is in line with our findings, i.e. PGC1α reduction and HIF1α promotion upon SOX2 silencing.

## Conclusions

In conclusion, with this study we would propose a thigh correlation between SOX2 expression and OxPhos metabolism in melanoma cells, under a condition of reduced HIF1α expression. Oxidative metabolism might be of a crucial importance for melanoma progression. Indeed, cancer cells may take advantage of this metabolic reprogramming toward OxPhos contributing to the development of an aggressive tumor phenotype endowed with an enhanced drug resistance and metastatic ability [48].

## Additional file

Additional file 1: **Figure S1.** Growth curves of melanoma cells with SOX2 depletion or over-expression under standard and acidic condition. **Figure S2.** Western blotting of a panel of glycolysis- and OxPhos-related proteins after SOX2 silencing and over-expression in melanoma cells. (PDF 359 kb)

## Abbreviations

2-DG: 2-Deoxiglucose
ATP5A1: ATP Synthase F1 Subunit Alpha
BS: binding site
COX4I: cytochrome c oxidase subunit 4 isoform 1
COX5B: cytochrome c oxidase subunit 5B
Cyt-c: cytochrome-c
GLUTs: glucose transporters
HIF1α: hypoxia-inducible factor α
HK2: hexokinase isoform 2
LDH-A: lactate dehydrogenase A
MCTs: monocarboxylate transporters
OxPhos: oxidative phosphorylation
PDK1: pyruvate dehydrogenase kinase 1
PDP2: pyruvate dehydrogenase phosphatase 2
PGC1α: peroxisome proliferator-activated receptor gamma coactivator 1-α
SOX2: sex-determining region Y (SRY)-Box2
TCA: tricarboxylic acid cycle
TSS: transcription starting site
