# Supplementary material for: SOX2 as a novel contributor of oxidative metabolism in melanoma cells
Source: Cell Commun Signal. 2018 Nov 22;16:87. doi: 10.1186/s12964-018-0297-z (PMC6249961; doi:10.1186/s12964-018-0297-z)
Supplement: Supplementary file 1 — Figure S1. Growth curves of melanoma cells with SOX2 depletion or over-expression under standard and acidic condition. Figure S2. Western blotting of a panel of glycolysis- and OxPhos-related proteins after SOX2 silencing and over-expression in melanoma cells. (PDF 359 kb) [file 12964_2018_297_MOESM1_ESM.pdf]

Additional file 1

SOX2 as a novel contributor of oxidative metabolism in melanoma cells.

Elena Andreucci[1]*, Silvia Pietrobono[2]*, Silvia Peppicelli[1], Jessica Ruzzolini[1], Francesca Bianchini[1], Alessio Biagioni[1], Barbara Stecca[2] and Lido Calorini[1,3].

[1]Department of Clinical and Experimental Biomedical Sciences “Mario Serio”, Section of Experimental Pathology and Oncology, University of Florence, Florence, Italy. [2]Core Research Laboratory, Institute for Cancer Research and Prevention (ISPRO), Florence, Italy. [3]Center of Excellence for Research, Transfer and High Education DenoTHE University of Florence, Florence, Italy.

* These authors contributed equally to this work.

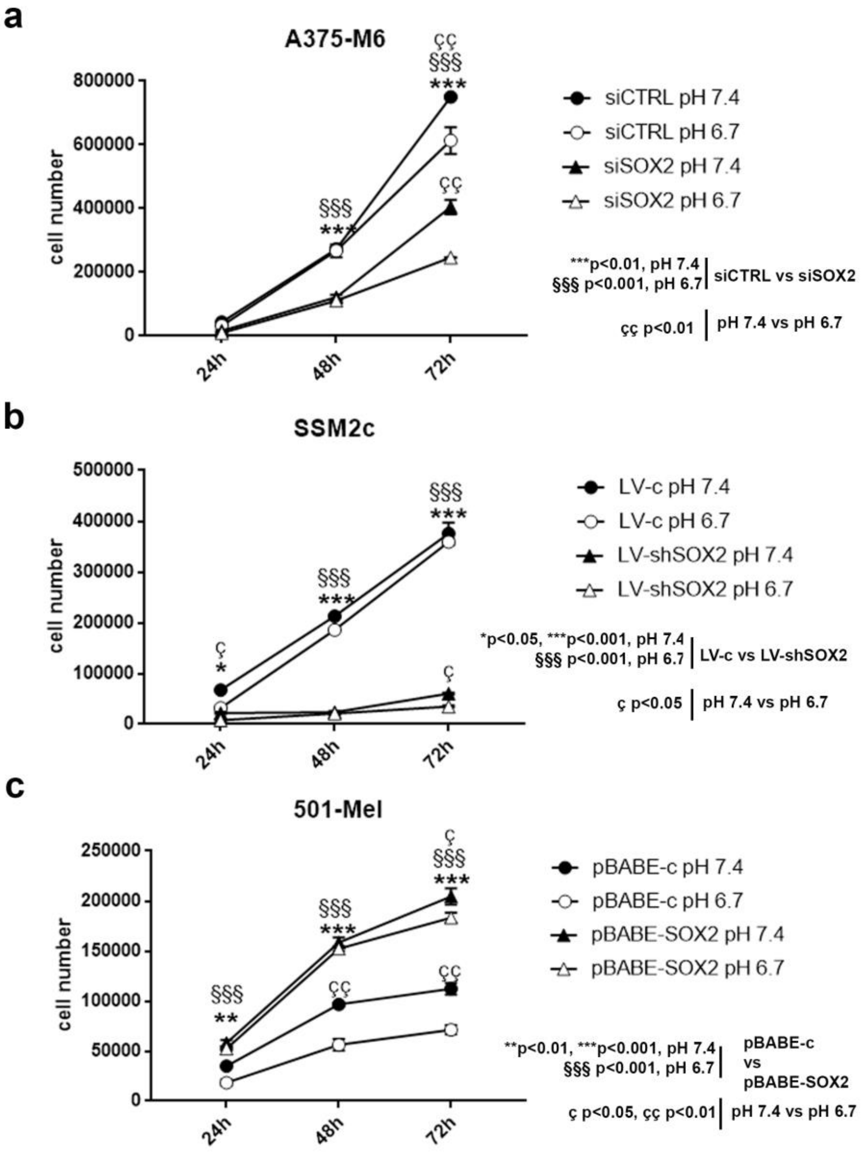

Figure S1 Growth curves of melanoma cells with SOX2 depletion or over-expression under standard and acidic condition. Proliferation of SOX2-depleted A375-M6 (a) and SSM2c (b), and SOX2-overexpressed 501-Mel (c) grown for 24, 48 and 72 hours at pH 7.4 and 6.7. GraphPad Prism software, Two-way ANOVA, N=3.

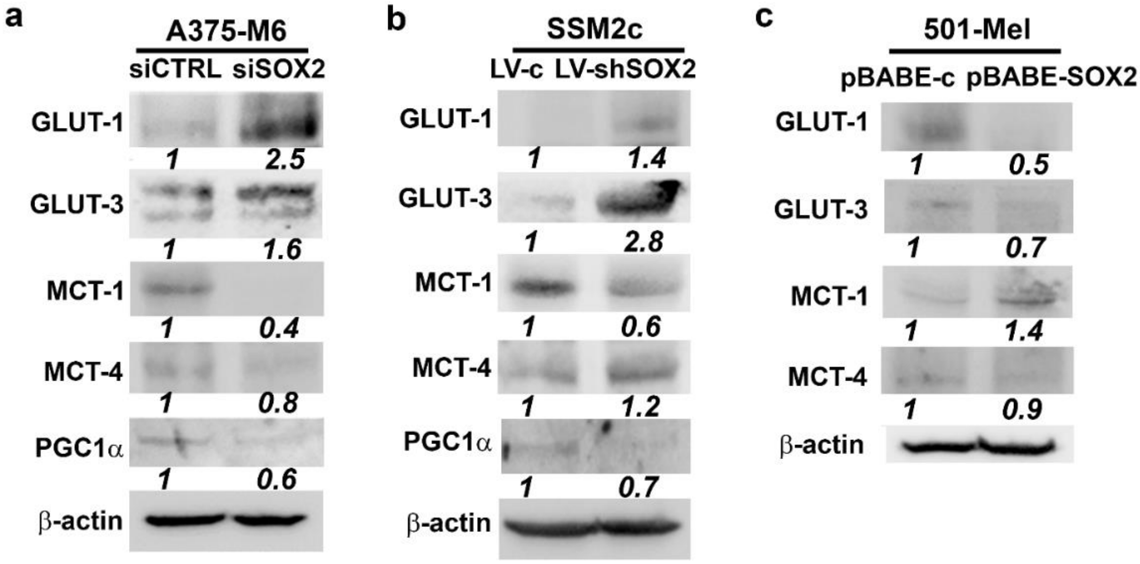

Figure S2 Western blotting of a panel of glycolysis- and OxPhos-related proteins after SOX2 silencing and over-expression in melanoma cells. a) Western blot of GLUT-1 ($p<0.01$), GLUT-3 ($p<0.05$), MCT-1 ($p<0.01$), MCT-4 (ns) and PGC1a ($p<0.05$) in A375-M6 siCTRL and siSOX2 at pH 7.4. T-test, N=3. b) Western blot of GLUT-1, GLUT-3, MCT-1, MCT-4 and PGC1a in SSM2c LV-c and LV-shSOX2 at pH 7.4. $p<0.05$, T-test, N=3. c) Western blot of GLUT-1 ($p<0.01$), GLUT-3 ($p<0.05$), MCT-1 ($p<0.01$), and MCT-4 (ns) in 501-Mel pBABE-c and pBABE-SOX2 at pH 7.4. T-test, N=3. Quantification of protein expression is shown in italic.
